# Supplementary material for: Defining the Transcriptional and Post-transcriptional Landscapes of Mycobacterium smegmatis in Aerobic Growth and Hypoxia
Source: Front Microbiol. 2019 Mar 26;10:591. doi: 10.3389/fmicb.2019.00591 (PMC6448022; doi:10.3389/fmicb.2019.00591)
Supplement: Supplementary file 2 [file Data_Sheet_1.docx]

**Supplementary Figures**

**Supplementary Figure 1. Hypoxia model similar to the Wayne model .** Cultures were grown in sealed flasks to produce a gradual reduction in oxygen. Samples were taken at 15 (S1) and 24 (S2) hours after bottles were sealed. For control, cultures were sampled at an OD = 0.8.

**Figure S2. Construction of 5’-end-directed libraries. A)** RNA samples were split in two parts and treated differentially. RNA for Library 1 (converted) was treated with RPPH to convert triphosphates in monophosphates, allowing the capture of 5’ end that are primary transcripts or cleaved RNAs. RNA for Library 2 (non-converted) was mock-treated, allowing the capture of cleaved transcripts. **B)** Workflow of 5’-end-directed libraries. After RPPH or 5’ polyphosphatase treatment, adapter SSS392 (TCCCTACACGACGCTCTTCCGAUCU) was ligated to the 5’ monophosphate ends (1). Then, RNA was fragmented by heating at 85°C for 6 min (log phase experiment) or at 94°C for 11 min (hypoxia experiment) (2) and first strand cDNA synthesis was carried out using the degenerate primer SSS397 (CTGGAGTTCAGACGTGTGCTCTTCCGATCTNNNNNN) (3). RNA was then degraded and DNA was amplified using universal adapter sequence SSS398 (AATGATACGGCGACCACCGAGATCTACACTCTTTCCCTACACGACGCTCTTC) and primers bearing Illumina indexes (4). Adapter-bearing products were PCR-amplified using outer primers SSS401 (AATGATACGGCGACCACCGAGATC) and SSS402 (CAAGCAGAAGACGGCATACGAGAT) to enrich for full-length fragments. 4 (log phase experiment) or 16 (hypoxia experiment) PCR cycles were performed (5). Finally, libraries were sequenced using Illumina technology (6).

**Figure S3. Workflow for noise filtering and TSS prediction in the different datasets.** Normoxia refers to the control (log phase) used in the hypoxia experiment.

**Supplementary Figure 4. TSSs identified in the different datasets.** Dataset 1: exponential phase (5,774 TSSs), Dataset 2: Normoxia (4,736 TSSs).

**Figure S5. Workflow used for TSS classification.** A complete scheme of the procedure used to classify TSSs is shown. TSSs located within 0-500 nt upstream of an annotated coding sequence were classified as pTSSs. TSSs located within annotated coding sequences were classified as iTSSs. iTSSs located within the first 25% of an annotated coding sequence were subclassified as N-iTSSs. When a gene lacked a pTSS, had an N-iTSS, and had an in-frame start codon downstream of the N-iTSS and within the first 30% of the coding sequence, the start codon of the gene was re-annotated. aTSSs (TSSs located on the antisense strand of a coding sequence, 5’ UTR, or 3’ UTR) and oTSSs (TSSs not belonging to any of the above-mentioned categories) were assigned as described in Figure 1D and Materials and Methods.

**Figure S6. Distribution of antisense TSSs.** The 1,006 aTSSs were classified according to their positions in 5’ UTRs, 3’ UTRs, and CDSs (coding sequences).

**Supplementary Figure 7. Cleavage sites distribution within genes according to coding sequence** **context.** The number of cleavage sites according to the relative position in the coding sequence is represented considering **A)** only coding sequences whose downstream gene is in the same strand, **B)** only coding sequences whose downstream gene is in the opposite strand (convergent), and **C)** only genes having a downstream gene transcribed as an operon. The CS distribution is significantly different between graphics A and B (*p-*value <0.0001, Kolmogorov Smirnov D test).

**Figure S8. Validation of a medium confidence pTSS. A)** Constructs used to validate the medium confidence pTSS of MSMEG_0063 were cloned into pJEB402 plasmid and integrated in the L5 site in the genome of an *M. smegmatis* strain lacking msmeg_0062-msmeg_0066. The *WT promoter* construct has the wildtype promoter region; *Δpromoter* has a deletion of the region upstream of the predicted pTSS; and *mutated promoter* has a replacement of two bases (red asterisks) in the -10 promoter region (underlined sequence). **B)** 1% agarose gel showing the 5’ RACE amplification products. The red arrows indicate the band corresponding to the predicted pTSS. At the bottom is indicated whether the RNA samples were treated with pyrophosphohydrolase (RPPH) prior to adapter ligation and whether cDNA synthesis with reverse transcriptase (RT) was performed. PCR control: water.

**Supplementary Figure 9. Gene expression levels in RNAseq expression libraries in hypoxia.** Changes in transcript levels were obtained by DEseq analysis, comparing each indicated condition to the control experiment. Genes upregulated (245 or 266 at 15 or 24 h, respectively) and downregulated 106 or 158 at 15 or 24 h, respectively) with a fold change ≥2 and a corrected p value ≤ 0.05 are highlighted in green and red, respectively. The triangle indicates expression of MSMEG_5244 (*dosR*) gene.

**Supplementary Figure 10. Correlation between expression data and 5’ end-directed libraries data in hypoxia.** The *X* axis represents the Log_2_ of the fold change in the expression libraries from hypoxia/normoxia datasets and the *Y* axis represents the Log_2_ of the fold change in the peak height in hypoxia/normoxia 5’end-directed libraries. The analysis was done for hypoxia at 15 hours **(A)** and 24 hours **(B)**. Genes having only one pTSS were used. The correlation is significant in both cases, with a *p-*value <0.00001.

**Supplementary Figure 11. Changes in RNA cleavage within coding sequences in hypoxic conditions.** The number of cleavage events within each coding sequence was compared through the different conditions. The Log_2_ of the ratio of the number of cleavages in hypoxia/control are shown. Each dot represents a specific gene. **A)** Hypoxia 15 hours, **B)** Hypoxia 24 hours.
